# Traffic-Related Air Pollution and Incident Type 2 Diabetes: Results from the SALIA Cohort Study

**DOI:** 10.1289/ehp.0901689

**Published:** 2010-05-27

**Authors:** Ursula Krämer, Christian Herder, Dorothea Sugiri, Klaus Strassburger, Tamara Schikowski, Ulrich Ranft, Wolfgang Rathmann

**Affiliations:** 1 Institut für Umweltmedizinische Forschung (IUF), Leibniz Center at Heinrich Heine University Düsseldorf, Düsseldorf, Germany; 2 Institute for Clinical Diabetology and; 3 Institute of Biometrics and Epidemiology, German Diabetes Center, Leibniz Center for Diabetes Research at Heinrich Heine University Düsseldorf, Düsseldorf, Germany

**Keywords:** air pollution, cohort study, inflammation, traffic, type 2 diabetes

## Abstract

**Background:**

Cross-sectional and ecological studies indicate that air pollution may be a risk factor for type 2 diabetes, but prospective data are lacking.

**Objective:**

We examined the association between traffic-related air pollution and incident type 2 diabetes.

**Design:**

Between 1985 and 1994, cross-sectional surveys were performed in the highly industrialized Ruhr district (West Germany); a follow-up investigation was conducted in 2006 using data from the Study on the Influence of Air Pollution on Lung, Inflammation and Aging (SALIA) cohort.

**Participants:**

1,775 nondiabetic women who were 54–55 years old at baseline participated in both baseline and follow-up investigations and had complete information available.

**Materials and Methods:**

Using questionnaires, we assessed 16-year incidence (1990–2006) of type 2 diabetes and information about covariates. Complement factor C3c as marker for subclinical inflammation was measured at baseline. Individual exposure to traffic-related particulate matter (PM) and nitrogen dioxide was determined at different spatial scales.

**Results:**

Between 1990 and 2006, 87 (10.5%) new cases of diabetes were reported among the SALIA cohort members. The hazards for diabetes were increased by 15–42% per interquartile range of PM or traffic-related exposure. The associations persisted when different spatial scales were used to assess exposure and remained robust after adjusting for age, body mass index, socioeconomic status, and exposure to several non–traffic-related sources of air pollution. C3c was associated with PM pollution at baseline and was a strong independent predictor of incident diabetes. Exploratory analyses indicated that women with high C3c blood levels were more susceptible for PM-related excess risk of diabetes than were women with low C3c levels.

**Conclusions:**

Traffic-related air pollution is associated with incident type 2 diabetes among elderly women. Subclinical inflammation may be a mechanism linking air pollution with type 2 diabetes.

**Relevance to clinical practice:**

Our study identifies traffic-related air pollution as a novel and potentially modifiable risk factor of type 2 diabetes.

Particulate matter (PM) air pollution is associated with an increased risk for cardiovascular events (Miller et al. 2007; Pope et al. 2004). There is compelling evidence that people with diabetes are more vulnerable to cardiovascular health effects associated with PM air pollution (O’Neill et al. 2005; Whitsel et al. 2009). Findings from a study conducted by Brook et al. (2008) have suggested that several of the biological pathways that have been linked to air pollution and cardiovascular disease (including systemic oxidative stress and low-grade inflammation) may also promote type 2 diabetes.

Recently, a cross-sectional study indicated higher diabetes prevalence among women who were exposed to higher levels of nitrogen dioxide (NO_2_), a marker of traffic-related air pollution (Brook et al. 2008). Another recent study demonstrated that ambient fine PM induces insulin resistance and an increase in visceral adiposity in mice (Sun et al. 2009). Subclinical inflammation has been postulated as a mediator between air pollution and cardiometabolic risk and has also been linked with impaired glucose metabolism (Kempf et al. 2008; Kolb and Mandrup-Poulsen 2005). Thus, an association between air pollution and type 2 diabetes appears conceivable and is biologically plausible. However, data from prospective studies on air pollution and incident type 2 diabetes are lacking.

We investigated the association between traffic-related air pollution and incident type 2 diabetes in the prospective Study on the Influence of Air Pollution on Lung, Inflammation and Aging (SALIA) cohort study of middle-aged women in Germany (Gehring et al. 2006; Schikowski et al. 2005, 2007). We focused on traffic-related air pollution, because it represents a major source of air pollution, particularly in urban areas. SALIA comprises female participants from the highly industrialized Ruhr district and two rural reference counties and has been used before to demonstrate associations between traffic-related air pollution and mortality and respiratory diseases (Gehring et al. 2006; Schikowski et al. 2005, 2007).

Furthermore, we investigated whether subclinical inflammation might influence susceptibility to traffic-related air pollution and tested whether the association between air pollution and diabetes might depend on elevated serum concentrations of complement factor C3c at baseline. C3c is a cleavage product of the complement factor C3, one of the most abundant complement proteins in the circulatory system. C3 is produced mainly in the liver, and systemic levels increase in infection and inflammation, although to a lesser extent than acute-phase proteins such as C-reactive protein (Moshage 1997; Ritchie et al. 2004). High levels of C3 in the circulation are found in obesity and have been implicated in the risk for cardiovascular disease (Engström et al. 2007; Hernández-Mijares et al. 2007). C3c represents a surrogate marker for subclinical inflammation and has been shown to be a risk factor for diabetes (Shima et al. 1999). It was measured at study baseline and showed an association with air pollution (Stiller-Winkler et al. 1989).

## Materials and Methods

### Study design and population

The SALIA cohort study is based on consecutive cross-sectional surveys that were performed between 1985 and 1994 as part of the Environmental Health surveys in North-Rhine Westphalia (West Germany). Seven different study areas from the highly industrialized Ruhr district [Dortmund (1985 [Dortmund (1990), Duisburg (1990), Essen (1990), Gelsenkirchen (1986 Gelsenkirchen (1990), and Herne (1986)] were chosen to represent a range of polluted areas with high-traffic load as well as steel and coal industries. Additionally two nearby nonindustrialized towns [Borken (1985, 1986, 1987, 1990, 1993, 1994) and Dülmen (1985 and Dülmen (1986)] were chosen as lower polluted areas. All women 54–55 years old who were living in the study region were asked to participate; a total of 4,874 women (70%) participated in the study (Schikowski et al. 2005). Gehring et al. (2006) conducted a mortality follow-up of these women in 2003.

In 2006, we performed a questionnaire follow-up and invited all women who were still living and who had addresses available (*n* = 4,027). A follow-up clinical examination in a randomly selected subgroup was performed in 2008–2009. We obtained written informed consent from all study participants. The study was approved by the ethics committee of the Ruhr University in Bochum (Germany).

### Baseline examination

The baseline questionnaire included items on symptoms and diagnoses of respiratory diseases [Fragebogen der Querschnittstudie 55jähriger Frauen (Ministerium für Umwelt, Raumordnung und Landwirtschaft des Landes Nordrhein-Westfalen 1993)]. We also asked about single-room heating with fossil fuels and occupational exposure (dust and extreme temperatures). Socioeconomic status was stratified into two categories by the maximum period of education achieved by either the women or their husbands (< 10 years vs. ≥ 10 years). Women were also grouped according to their smoking habits as never smokers, passive smokers (home, workplace), past smokers, or current smokers (further categorized by < 15, 15–30, and > 30 pack-years).

Height and weight were measured under standardized conditions. Nonfasting serum samples were collected and stored in the central laboratory at the Hygiene Institute (Düsseldorf University, Germany) at − 70°C for a maximum period of 3 months prior to analysis. The complement factor 3 cleavage product C3c was determined in a central laboratory at the Hygiene Institute (before 1990 using immunoprecipitation methods and after 1990 with nephelometry; all analyses with C3c data in this study were adjusted for the two different analytic methods using regression analysis (Fink et al. 1989; Stiller-Winkler et al. 1989).

### Follow-up questionnaire and investigation

In 2006, study participants received a follow-up questionnaire [a modified version of the baseline questionnaire that included additional questions on diabetes (age of onset, treatment) and other diseases] in the mail from the local health departments where the baseline investigation already had taken place. The health authorities from Herne could not participate in 2006, so addresses from Herne were missing. The self-administered questionnaire included the questions from the baseline questionnaire and, in addition, several items on cardiometabolic diseases. For example, we asked whether the participants had ever been diagnosed by a physician with hyperlipidemia, myocardial infarction, stroke, or diabetes. With respect to diabetes, the year of diagnosis, antidiabetic treatment, and type of medication were asked. The incidence of physician-diagnosed diabetes after 1990 was chosen as the main outcome (16-year incidence). The beginning of antidiabetic treatment was not documented. The vast majority of incident diabetes cases in this age group can be assumed to have type 2 diabetes. In the case of Borken (control region), where some of the baseline examinations were performed in 1993 and 1994, we counted only diagnoses after these dates as cases.

In the 2008–2009 clinical examination, the same questions on diabetes as in the 2006 questionnaire were asked in an interview. These data were used to test the reliability of the questionnaire results.

### Assessment of exposure to ambient air pollution

We used four methods with different spatial scales to characterize exposure of the women [see Supplemental Material (doi:10.1289/ehp.0901689)]. First, data from monitoring stations maintained by the State Environment Agency covering the Ruhr area in an 8-km grid were used to reflect broad-scale spatial variation in air quality. The 5-year means of 1986–1990 at the measurement stations, which were located nearest to the home addresses of the women, were used. Second, we assessed the exposure to motor vehicle exhaust (1-km grid) by using emission inventories provided by the State Environment Agency. The agency estimated the annual mass of PM and NO_2_ emitted per square kilometer from road traffic. We used the emission inventory from 1994 to characterize emissions in the square kilometer where the women lived. Third, we applied land-use regression models to assign estimated NO_2_ and soot concentrations from traffic-related sources to the residential address of each individual (Brauer et al. 2003; Hochadel et al. 2006). Briefly, the models were based on a 1-year measurement program conducted in 2002. Concentrations of NO_2_, fine PM [aerodynamic diameter ≤ 2.5 μm (PM_2.5_)], and filter absorbance of PM_2.5_ were measured at 40 points in two regions (Duisburg, Borken) based on 14-day samples for each season (spring, summer, fall, winter) and site. The models could explain 92% and 87% of the variance in NO_2_ and PM_2.5_ absorbance, respectively. Modeled NO_2_ and PM_2.5_ absorbance (soot) were then applied to the women’s addresses in the year 1990. Fourth, exposure was characterized by the distance from home address at baseline investigation to the next major road with > 10,000 cars per day (highly exposed: distance to street < 100 m).

During 1985–1995 a steep decline in air pollution, especially in PM pollution, took place in Germany. However, this did not affect the spatial pattern of traffic-related pollution in our investigation area. The correlations were fairly stable over time [see Supplemental Material (doi:10.1289/ehp.0901689)]. The ranking of traffic-related exposure remained the same throughout the follow-up period of 16 years. We therefore assumed that measurements performed after the end of the baseline investigation still reflected the pattern of pollution the women were exposed to before 1990 and are valid to determine the association between pollution pattern and the incidence of diabetes. Because of the steep decline of air pollution, we used air pollution data from 1990 and started counting incident cases of diabetes from 1990 to 2006 instead of using data from the individual time points of the baseline investigation. Therefore, length of follow-up is most likely not confounded by exposure at baseline.

### Statistical analyses

Data are given as proportions for categorized variables or means and SD or geometric means and geometric SDs for continuous variables with normal or with log-normal distribution, respectively. Chi-square tests and *F*-tests were used to detect significant differences between groups. We analyzed the association of type 2 diabetes incidence with ambient air pollution exposure using Cox regression analyses and accounted for ties using the exact method. Incident cases were women who reported being newly diagnosed with diabetes by a physician after 1990. Hazard ratios (HRs) with corresponding 95% confidence intervals (CIs) were estimated for increases of 1 interquartile range (IQR; calculated from the distribution of the whole sample) in PM or NO_2_ exposure and for residential address < 100 m to a busy road. The level of significance was set as 0.05. Age, body mass index (BMI), socioeconomic status, current smoking, exposure to environmental tobacco smoke (ETS), occupational exposure to temperature (heat and cold) and dust, as well as heating with fossil fuels were included as covariates in all models to adjust for potential confounding. BMI as covariate in regression analyses was calculated as the mean of BMI at baseline and at follow-up. The correlation between both these determinations was high (*r* = 0.77). In exploratory analyses, the study population was subdivided along the median of C3c serum levels (100.40 mg/dL) of all participants with complete data on C3c, all covariables at baseline and available follow-up information; HRs (95% CI) were calculated separately for both C3c subgroups. Whether or not C3c levels modified the association of the exposure variables with type 2 diabetes risk was tested by including an interaction term in the models. All statistical analyses were carried out with SAS for Windows (version 9.1; SAS Institute Inc., Cary, NC, USA). ArcView (version 9.1; ESRI, Redlands, CA, USA) was used for the geographic data.

## Results

### Characteristics of study population

The study was based on 1,775 women who participated in both baseline and follow-up investigations, who had data for all covariables, and who had no diagnosis of diabetes before 1990. Study participants differed from nonparticipants who were still alive at follow-up (*n* = 1,933) and participants who died during follow-up (*n* = 585) mainly by a higher level of education, a slightly lower BMI, a lower prevalence of hypertension, and lower C3c levels [see Supplemental Material, Table 1 (doi:10.1289/ehp.0901689)].

Of the women who reported no diagnosis of diabetes before 1990, 187 (10.5%) reported being newly diagnosed with diabetes after 1990. In 1990, 54 women already had a diagnosis of diabetes and were therefore excluded from the analysis. Table 1 shows the characteristics of women with newly diagnosed diabetes and those without a diagnosis of diabetes. Those with incident diabetes were slightly older, had a higher BMI, had higher C3c values, suffered more often from hypertension, and more frequently had a history of myocardial infarction or stroke (Table 1). Furthermore, women who had been diagnosed with diabetes were less educated, heated their rooms more often with ovens using fossil fuels, had higher workplace dust exposure, and were more likely to be smokers than were those who had never been diagnosed with diabetes. Slightly more women with incident diabetes (20.9%) changed their address than did women without diabetes (15.4%). The majority of these women did not change their post-code area when they moved.

We evaluated the reliability of the questionnaire answers concerning a diabetes diagnosis. In the follow-up interview in 2008–2009, 386 women answered the questions pertaining to diabetes. Two women reported a diagnosis of diabetes in the 2006 questionnaire but not in the interview. In the interview, two women who had claimed an onset before 1990 now reported an onset after 1990. Therefore, 382 replies in the interview regarding the diagnosis of diabetes between 1990 and 2006 were concordant with the questionnaire from 2006 (99% concordance).

### Exposure to ambient air pollution

Exposure to ambient air pollution was higher among women who developed type 2 diabetes in the 16-year follow-up (Table 2). This finding was demonstrated for all chosen indicators and for all spatial scales. It is noteworthy that mean PM emissions at the place of residency of women who lived near busy streets and who had a low-education level was significantly higher than those of women with a higher education level who lived near busy streets (2.01 t/year/km^2^ compared with 1.83 t/year/km^2^). Such a socioeconomic gradient did not exist for women who lived further away from busy streets.

### Association between ambient air pollution and diabetes

All crude and adjusted HRs for the associations between exposure to ambient air pollution and incident type 2 diabetes were > 1.0 and were statistically significant except exposure to broad-scale PM_10_ (Table 3). The risk of incident type 2 diabetes was increased by 15% (95% CI, 4–27) per 1 IQR of traffic-related PM exposure (*p*-value = 0.009). The associations between exposure and incident diabetes depended on exposure metric and were highest for the metric with the highest spatial resolution. For each exposure metric, the associations with NO_2_ were stronger than (or at least equal with) PM: The HRs for NO_2_ were increased by 15% (95% CI, 4–27) to 42% (95% CI, 16–73) (*p-*values < 0.001–0.005).

In a sensitivity analysis, we performed a stratified Cox regression with two strata, namely, urban and rural living. The urban areas are all in the Ruhr area and of very similar structure and type. The estimated effects in the urban areas were nearly unchanged from the estimates for all areas combined and were all significant, whereas the effect estimates were greater in the rural area but not significant. We also adjusted for urban and rural living, which had hardly any impact on the estimates. This result further indicates that the associations are not due only to confounding by factors related to urban and rural living. As additional sensitivity analysis, we fitted regression models that included fixed effects for the nine different locations (seven towns, including two towns with two different study areas). This type of analysis was not possible for exposure based on monitoring stations, because only one monitoring station per location was available. The HRs were slightly smaller than those without adjusting for location. They all remained positive, but only distance < 100 m from busy road and NO_2_ from land-use regression remained statistically significant (data not shown).

We did a further sensitivity analysis and conducted Cox regression analysis that included only women who did not change their addresses during follow-up (*n* = 1,492). The HRs for the single-exposure variables were not altered systematically, and overall the results did not change (data not shown). A change of residency was fairly seldom; thus, assigning pollution exposure based on residence at the beginning of the study probably introduced no systematic bias.

In further analyses, we adjusted for hypertension and change in BMI or excluded persons with coronary heart disease at baseline or follow-up and found hardly any change in the results (data not shown).

Women with low education were more exposed to PM pollution when living near a busy road than women with high education. Therefore, we included an interaction term (education*living near a busy road) in the regression models. Type 2 diabetes incidence among women with low education more than doubled when they lived near a busy road (adjusted HR = 2.54; 95% CI, 1.31–4.91; *p* = 0.006) compared with those women with low education who did not live near a busy road. The corresponding adjusted HR (95% CI) for women with high education near a busy road was 0.92 (0.58–1.47) compared with those who did not live near a busy road (*p* for interaction = 0.0141).

HRs for the associations of the included covariates (listed in Table 3) with incident type 2 diabetes are given in the Supplemental Material, Table 2 (doi:10.1289/ehp.0901689). After adjusting for all confounders, single-room heating with fossil fuels and former smoking as indicators of non–traffic-related air pollution were significantly associated with increased diabetes risk. Age, education, and workplace exposure (dust or fumes or extreme temperatures), which had been significant predictors in univariate analyses, lost significance after adjusting for all other covariates.

### Ambient air pollution, subclinical inflammation, and diabetes

Serum concentrations of C3c at baseline were significantly associated with PM_10_ levels (1985–1989) after adjusting for age, BMI, heating with fossil fuels, workplace exposure to dust or fumes, extreme temperatures, smoking, and education (linear regression with log-transformed values; ratio of means and 95% CI) for a 1-IQR PM_10_ increase: 1.09 (1.06–1.12, *p* < 0.001). This association was comparable in the two nonresponder groups (death before 2006: 1.13 (1.06–1.20), *n* = 284; *p* < 0.001; alive, but no participation in 2006: 1.09 (1.06–1.13), *n* = 890; *p* < 0.001). Moreover, elevated levels of C3c were associated with an increased diabetes risk: The adjusted HR (95% CI) for an increase in C3c by 10 mg/dL was 1.12 (1.05–1.18) (*p* < 0.001).

In exploratory analyses, we subdivided the study population according to their median C3c serum level at baseline and repeated the calculation of adjusted HRs and 95% CIs for incident type 2 diabetes (from Table 3) in both subgroups with low and high C3c at baseline (*n* = 617 and *n* = 634, respectively; Table 4). For most exposure variables, we found evidence for interaction between C3c at baseline and risk for type 2 diabetes. Stratified analyses showed that an elevated risk for type 2 diabetes was present only for women with high C3c at baseline, whereas for women with low C3c at baseline, no association between exposure to ambient air pollution and incident type 2 diabetes could be revealed.

## Discussion

To the best of our knowledge, this is the first prospective, population-based study that showed a statistically significant association between traffic-related air pollution and incident type 2 diabetes. This association persisted when four different spatial scales were used to assess exposure levels and remained robust after adjustment for confounders including BMI, socioeconomic status, and exposure to several indoor sources of air pollution.

Currently, data on air pollution and type 2 diabetes risk are scarce. The first evidence came from an ecological study that found a significant relationship between total state air emissions for all industries and prevalence of diabetes in the United States (Lockwood 2002). More recently, a cross-sectional study in Canada suggested a positive association between NO_2_ exposure as a marker of traffic-related air pollution and type 2 diabetes among women (Brook et al. 2008). This study reported an increase in the adjusted odds of diabetes by nearly 17% for an increase in NO_2_ of 1 IQR, which is in line with our findings (adjusted HR: 1.15–1.42, depending on NO_2_ assessment). Furthermore, an experimental study in mice showed that fine PM in the air (PM_2.5_) induced insulin resistance, increased adipose tissue macrophages, altered the balance between macrophage subtypes in adipose tissue, and raised systemic levels of immune mediators that have previously been implicated in the development of type 2 diabetes (Sun et al. 2009). More evidence for such an association among humans comes from a recent population-based cross-sectional study of 10- to 18-year-old children in Isfahan, Iran (Kelishadi et al. 2009). The city of Isfahan currently faces a huge increase in air pollution from rapid industrial development and heavy traffic of motor vehicles. Air pollution (PM_10_, carbon monoxide, pollutant standard index) was independently associated with insulin resistance (homoeostasis model assessment model), a hallmark of type 2 diabetes, and low-grade inflammation in children and young adults, even after adjusting for BMI, waist circumference, dietary intake, and physical activity (Kelishadi et al. 2009). Collectively, these data suggest that air pollutants may increase the risk of type 2 diabetes by impairing insulin sensitivity and point toward inflammatory processes as a mechanistic link.

C3c may play a role in the development of air pollution–related insulin resistance and type 2 diabetes, because epithelial cells and macrophages at the airway surface produce multiple components of the complement system that exert a central role in innate immunity (Cole et al. 1983; Strunk et al. 1988). Airway exposure to PM activates C3 in mice (Walters et al. 2002), and C3c is a stable cleavage product of C3 that can be used as an indicator of the activation of the innate immunity. C3 is of interest for two reasons. First, we and others previously demonstrated higher C3c levels in inhabitants of areas with a higher degree of air pollution (Shima et al. 1999; Stiller-Winkler et al. 1989), which is in accordance with data from murine models (Walters et al. 2002). Second, elevated C3 levels were associated with incident type 2 diabetes in our study population, which confirms results from a population-based cohort study in Sweden (Engström et al. 2005). It is biologically plausible that air pollution activates the innate immunity in the lung and that this immune activation then spreads to other parts of the body and becomes apparent in chronically elevated levels of pro-inflammatory biomarkers in the circulation that have a negative impact on insulin sensitivity and beta-cell function. However, it should be noted that C3c represents only one of many activities of the immune system. Measurement of additional inflammatory markers would be necessary to substantiate this hypothesis.

In addition to being part of the causal pathway between air pollution and insulin resistance or type 2 diabetes, it is also conceivable that a chronic activation of the immune system increases the susceptibility of individuals to air pollution. Our data are in line with this latter interpretation. It has been reported before that individuals with insulin resistance or type 2 diabetes, both of which are proinflammatory conditions, are more vulnerable to ambient PM exposure and show stronger associations between exposure and cardiovascular risk factors such as reduced heart rate variability and impaired vascular reactivity (O’Neill et al. 2005; Whitsel et al. 2009). This interpretation, which considers C3c (and subclinical inflammation in general) as effect modifier rather than a mechanistic factor downstream of particle exposure, raises the question of which other factors are determinants of immune activation. Our study was not designed to answer this question, but we hypothesize that many variables, including genetic predisposition, lifestyle components, and presence of inflammation-related subclinical comorbidities like atherosclerosis or nonalcoholic fatty liver disease, could be relevant contributors. Even if we cannot disentangle the relationship between air pollution, inflammation, and risk for type 2 diabetes, our findings appear intriguing, because they indicate the presence of subgroups in the population that are more or less vulnerable to the impact of air pollution. Therefore, our results may stimulate further research to better understand the interaction of inflammation and air pollution for the incidence of adverse health outcomes and for the identification of at-risk individuals.

We found slightly stronger associations with NO_2_ exposure than with PM-related exposure assessments. The main sources of NO_2_ are traffic related, whereas PM may also stem from industrial sources. Therefore, stronger associations with NO_2_ strengthen our view that traffic-related pollution is responsible for associations with incident diabetes.

Our study has several strengths that should be mentioned briefly. The association between traffic-related air pollution and incident type 2 diabetes was present when we used different indicators of traffic-related air pollution and different spatial scales. Moreover, the availability of relevant non–traffic-related indicators of air pollution allowed us to adjust for potentially confounding effects.

The main limitation of the study is the fact that type 2 diabetes was assessed by self-report only. The reliability of these reports, however, was high. An interview conducted 2 years after the questionnaire follow-up in 2006 showed 99% concordance. We also did not have glucose measurements. This may have led to outcome misclassification (underdiagnosis), but this is most likely unrelated to exposure. In theory, this misclassification might be related to exposure, for example, if women with cardiovascular diseases due to air pollution visited their physician more often and then had a diagnosis of diabetes. If this were true, we would expect stronger associations of pollution with cardiovascular outcomes than with diabetes, which was not the case. In our study, the association of pollution with incident diabetes was much stronger than the association with myocardial infarction, stroke, or hypertension. The adjusted association of physician-diagnosed hypertension with NO_2_, for instance, was 1.09 (95% CI, 0.93–1.27) compared with 1.49 for physician-diagnosed diabetes (data not shown). Another limitation is that our follow-up was not complete and that women with higher education were overrepresented. Because the incidence of diabetes was lower in higher-educated women, this may have led to an underestimation of diabetes incidence. The association with air pollution, however, is likely not confounded by selective participation because *a*) we adjusted for education in our analysis, and *b*) the association of the risk factor C3c with air pollution was the same in participating and nonparticipating women. Another limitation might be that C3c levels were available for only 70% of the cohort. Further inflammatory markers would be required to characterize more accurately the components of subclinical inflammation that are important in the relationship between air pollution and type 2 diabetes. Furthermore, data on diet, diabetes family history, and physical exercise were not available. However, we investigated the effect of BMI, which might also reflect differences in dietary habits and physical exercise. BMI was not positively associated with any of our exposure variables and therefore could not be a confounder. Interestingly, change in BMI was associated with air pollution, that is, the adjusted change in BMI was 0.4 kg/m² per IQR of NO_2_ exposure (land-use regression). However, the association between air pollution and incident diabetes remained unchanged even after including BMI change into the model. Finally, we had no data on antihypertensive medications or drugs for coronary heart disease available. However, as a sensitivity analysis, we additionally adjusted the analysis for hypertension and excluded persons with CHD at baseline or follow-up from the analysis. The results hardly changed. We therefore assume that the effects of these medications most likely did not confound our results.

Adjustment for the different locations in the models led to lower HRs than without adjustment, although they remained positive. Only distance < 100 m from busy road and NO_2_ from land-use regression remained statistically significant. However, we consider this an indication of overadjustment in the model, because air pollution and type of area are closely related. Additionally, we did a stratified Cox regression with two strata (urban and rural living) and did an additional analysis including urban and rural living as fixed effect in the model. The urban areas are of very similar structure and type. The effect estimates were nearly unchanged. This indicates that the associations are not due only to confounding by factors associated with urban or rural living, although we cannot preclude residual confounding by socioeconomic and lifestyle factors. We also cannot exclude that the association between diabetes incidence and living near a major road might be at least partially determined by other factors such as noise. Finally, the cohort comprised middle-aged women only, so the findings may not be representative for other groups of the population.

If confirmed, our study results have important implications for prevention of type 2 diabetes. About half of the world’s population now lives in urban areas, and many of the urban centers are expanding rapidly to megacities (Molina and Molina 2004). Air pollution, primarily from traffic, has become one of the most important problems of megacities. Vulnerable populations, such as those living in areas with low socioeconomic status, are disproportionately affected by air pollution (Younger et al. 2008). Our findings suggest a potential new pathway to reduce the risk of type 2 diabetes in the population by reductions in traffic-related air pollution.

Furthermore, our findings are interesting because numerous epidemiological studies have shown a higher prevalence of type 2 diabetes in urban areas compared with rural areas (Ramachandran et al. 1999; Sobngwi et al. 2004). This difference has been observed particularly in developing countries that have undergone a rapid transition from rural to urban lifestyle. It has been largely attributed to broad shifts in diet, physical activity, and obesity in urban areas (Popkin 1999). However, these lifestyle factors fail to completely explain the association with increased diabetes and obesity risk (Popkin 1999). Other environmental factors would also be expected to contribute to these striking differences, and further studies are needed to investigate the extent to which traffic-related air pollution determines the environmental health burden in cities worldwide.

Traffic-related air pollution is associated with increased risk to develop type 2 diabetes in the German SALIA cohort of women 54–55 years old. Subclinical inflammation may be one of the mechanisms that link air pollution with metabolic impairments. Further studies are needed to substantiate our novel finding and to better understand the contribution of chronic exposure to air pollution to excess risk of type 2 diabetes in residents of urban areas.

## Figures and Tables

**Table 1:** Characteristics of women in the SALIA study population stratified by type 2 diabetes status.^a^

Variable	No diabetes (*n* = 1,588)	Incident diabetes (*n* = 187)	*p-*Value
At baseline			
Age (years)	54.4 ± 0.70	54.6 ± 0.51	0.0003
BMI (kg/m^2^)^b^	26.7 ± 3.8	30.0 ± 4.5	< 0.0001
C3c (mg/dL)^b^,^c^	89.7 (1.32)	108.3 (1.31)	< 0.0001
Hypertension (%)	20.1	43.5	< 0.0001
Education < 10 years (%)^d^	19.8	27.3	0.0174
Single-room heating with fossil fuels (%)	16.5	27.3	0.0003
Workplace exposure			
Dust/fumes (%)^e^	9.0	12.8	0.0898
Extreme temperatures (%)^f^	6.4	10.2	0.0551
Smoking (%)			0.1184
Never, without ETS^g^	45.7	39.1	
Never, with ETS^g^	33.7	32.6	
Ex-smoker	8.1	12.8	
Current; 0–15 pack-years	5.0	4.8	
Current; > 15–30 pack-years	4.7	5.9	
Current; > 30 pack-years	2.8	4.8	

At follow-up			
Age (years)	71.0 ± 3.4	71.7 ± 2.8	0.0116
BMI (kg/m^2^)	26.5 ± 4.0	29.8 ± 4.9	< 0.0001
Hypertension (%)	50.2	72.4	< 0.0001
Hyperlipidemia (%)	42.1	49.7	0.0563
History of myocardial infarction (%)	3.2	9.7	< 0.0001
History of stroke (%)	3.5	7.6	0.0079
Change in address (%)	15.4	20.9	0.0524
Without change in postcode (%)	8.9	9.1	0.9463
With change in postcode (%)	6.4	11.8	0.0067

a Data are given as mean ± SD or proportions unless indicated otherwise; the proportion of missing values was < 10% for all variables except C3c [*n* = 466 missing (29%) among those without diabetes, 58 missing (31%) among those with diabetes].

b Measurements at day of baseline investigation; all other variables are based on questionnaires.

c Geometric mean (geometric SD).

d Maximum number of years of education of study participant or husband.

e Questionnaire-based information: self-reported long-lasting exposure at workplace to dust, gases, or fumes.

f Questionnaire-based information: self-reported long-lasting exposure at workplace to heat, chilliness, or wetness.

g ETS, environmental tobacco smoke exposure (workplace or home).

**Table 2:** Exposure of women in the SALIA study population to ambient air pollution, especially traffic-related air pollutants, assessed on four spatial scales and stratified by type 2 diabetes status.^a^

	No diabetes (*n* = 1,588)	Incident diabetes (*n* = 187)	All (*n* =1,775)	*p*-Value
Variable	P50 (P25–P75)^b^	P50 (P25–P75)	P50 (P25–P75)	Wilcoxon test
Monitoring stations				
PM_10_ (μg/m^3^)	46.9 (44.0–54.1)	48.6 (44.0–54.1)	46.9 (44.0–54.1)	0.0002
NO_2_ (μg/m^3^)	41.7 (23.3–48.2)	46.5 (23.3–48.2)	41.7 (23.3–48.2)	0.0003
Emission inventory				
PM from traffic (tons/year/km^2^)	0.54 (0.20–1.06)	0.66 (0.34–1.46)	0.54 (0.22–1.09)	0.0002
NO_2_ from traffic (tons/year/km^2^)	11.7 (5.2–23.6)	12.9 (8.2–31.4)	12.0 (5.4–24.4)	0.0022
Land-use regression				
Soot (10^−5^ m)	1.88 (1.66–2.05)	1.96 (1.72–2.14)	1.89 (1.67–2.06)	< 0.0001
NO_2_ (μg/m^3^)	33.9 (23.8–38.6)	36.7 (26.3–39.4)	34.5 (23.8–38.8)	0.0001
Distance < 100 m from busy road (%)^c^	15.6	17.7	—	0.4549^d^

a Five-year averages for the stationary sources were used. The proportion of missing values was < 10% for all variables.

b P50 (P25–P75): median and the 25th and 75th percentiles.

c More than 10,000 cars/day.

d *p*-Value χ^2^-test

**Table 3:** Association between incidence of type 2 diabetes (1990–2006) and exposure to ambient air pollution, especially traffic-related air pollutants, assessed on four spatial scales in a cohort of elderly women (SALIA study).

Variable	HR (95% CI) per IQR of exposure^a^		*p-*Value adjusted^b^
	Unadjusted	Adjusted^b^	
Monitoring stations			
PM_10_	1.64 (1.20–2.25)	1.16 (0.81–1.65)	0.4146
NO_2_	1.53 (1.20–1.95)	1.34 (1.02–1.76)	0.0377
Emission inventory			
PM from traffic	1.23 (1.12–1.35)	1.15 (1.04–1.27)	0.0087
NO_2_ from traffic	1.22 (1.11–1.34)	1.15 (1.04–1.27)	0.0052
Land-use regression			
Soot	1.28 (1.12–1.47)	1.27 (1.09–1.48)	0.0014
NO_2_	1.47 (1.22–1.77)	1.42 (1.16–1.73)	0.0006
Distance/education			
< 100 m from busy road/low^c^	2.32 (1.29–4.17)	2.54 (1.31–4.91)	0.0057^e^
< 100 m from busy road/high^d^	0.86 (0.55–1.36)	0.92 (0.58–1.47)	0.7379^e^

a The respective quartiles are given in Table 2.

b Adjusted for age, BMI, heating with fossil fuels, workplace exposure with dust/fumes, extreme temperatures, smoking, education [see Supplemental Material, Table 2 (doi:10.1289/ehp.0901689)].

c More than 10,000 cars/day; HR for the subgroup of women with low educational status.

d More than 10,000 cars/day; HR for the subgroup of women with high educational status.

e *p*_interaction_ = 0.0141.

**Table 4:** Association between the incident type 2 diabetes (1990–2006) and exposure to ambient air pollution, especially traffic-related air pollutants, assessed on four spatial scales in a cohort of elderly women (SALIA study) with high and low C3c: results of Cox regression analyses using a study sample of 1,251 subjects with complete information on all covariates and data of C3c concentrations.

	Adjusted^a^ HR (95% CI) per interquartile range of exposure^b^		
Variable	C3c < median (< 100.4 mg/dL) (*n* = 617)	C3c ≥ median (≥ 100.4 mg/dL) (*n* = 634)	*p*-Value (interaction)
Monitoring stations			
PM_10_	1.07 (0.70–1.64)	1.21 (0.70–1.64)	0.0088
NO_2_	0.92 (0.62–1.38)	1.29 (0.93–1.79)	0.0118
Emission inventory			
PM from traffic	0.91 (0.70–1.18)	1.24 (1.08–1.41)	0.0208
NO_2_ from traffic	0.85 (0.64–1.13)	1.24 (1.08–1.41)	0.0095
Land-use regression			
Soot	1.08 (0.88–1.32)	1.22 (1.02–1.47)	0.0064
NO_2_	1.03 (0.75–1.41)	1.31 (1.01–1.70)	0.0099
Distance/education			
< 100 m from busy road/low^c^	1.06 (0.14–8.24)	3.51 (1.50–8.23)	0.2796
< 100 m from busy road/high^d^	0.32 (0.08–1.31)	0.85 (0.42–1.70)	0.2155

a Adjusted for age, BMI, heating with fossil fuels, workplace exposure with dust/fumes, extreme temperatures, smoking, education [see Supplemental Material, Table 2 (doi:10.1289/ehp.0901689)].

b Respective quartiles are given in Table 2.

c More than 10,000 cars/day; estimates for women with low educational status.

d More than 10,000 cars/day; estimates for women with high educational status.
